# Early-Stage Repetitive Transcranial Magnetic Stimulation Altered Posterior–Anterior Cerebrum Effective Connectivity in Methylazoxymethanol Acetate Rats

**DOI:** 10.3389/fnins.2021.652715

**Published:** 2021-05-21

**Authors:** Huiling Guo, Yao Xiao, Dandan Sun, Jingyu Yang, Jie Wang, Huaning Wang, Chunyu Pan, Chao Li, Pengfei Zhao, Yanbo Zhang, Jinfeng Wu, Xizhe Zhang, Fei Wang

**Affiliations:** ^1^Department of Psychiatry, The First Affiliated Hospital of China Medical University, Shenyang, China; ^2^Early Intervention Unit, Department of Psychiatry, Affiliated Nanjing Brain Hospital, Nanjing Medical University, Nanjing, China; ^3^Functional Brain Imaging Institute of Nanjing Medical University, Nanjing, China; ^4^Department of Radiology, The First Affiliated Hospital of China Medical University, Shenyang, China; ^5^Key Laboratory of Magnetic Resonance in Biological Systems, State Key Laboratory of Magnetic Resonance and Atomic and Molecular Physics, National Center for Magnetic Resonance in Wuhan, Wuhan Institute of Physics and Mathematics, Innovation Academy for Precision Measurement Science and Technology, Chinese Academy of Sciences-Wuhan National Laboratory for Optoelectronics, Wuhan, China; ^6^Department of Psychiatry, Xijing Hospital, Fourth Military Medical University, Xi’an, China; ^7^School of Computer Science and Engineering, Northeastern University, Shenyang, China; ^8^Department of Psychiatry, Faculty of Medicine and Dentistry, The Neuroscience and Mental Health Institute (NMHI), University of Alberta, Alberta, AB, Canada; ^9^School of Biomedical Engineering and Informatics, Nanjing Medical University, Nanjing, China; ^10^Nanjing Brain Hospital, Nanjing Medical University, Nanjing, China

**Keywords:** methylazoxymethanol acetate, schizophrenia, repetitive transcranial magnetic stimulation, functional magnetic resonance imaging, Granger Causality Analysis, visual cortex, nucleus accumbens

## Abstract

The aim of the current resting-state functional magnetic resonance imaging (fMRI) study was to investigate the potential mechanism of schizophrenia through the posterior–anterior cerebrum imbalance in methylazoxymethanol acetate (MAM) rats and to evaluate the effectiveness of repetitive transcranial magnetic stimulation (rTMS) as an early-stage intervention. The rats were divided into four groups: the MAM-sham group, vehicle-sham group, MAM-rTMS group, and vehicle-rTMS group. The rTMS treatment was targeted in the visual cortex (VC) in adolescent rats. Granger Causality Analysis (GCA) was used to evaluate the effective connectivity between regions of interest. Results demonstrated a critical right VC–nucleus accumbens (Acb)–orbitofrontal cortex (OFC) pathway in MAM rats; significant differences of effective connectivity (EC) were found between MAM-sham and vehicle-sham groups (from Acb shell to OFC: *t* = −2.553, *p* = 0.021), MAM-rTMS and MAM-sham groups (from VC to Acb core: *t* = −2.206, *p* = 0.043; from Acb core to OFC: *t* = 4.861, *p* < 0.001; from Acb shell to OFC: *t* = 4.025, *p* = 0.001), and MAM-rTMS and vehicle-rTMS groups (from VC to Acb core: *t* = −2.482, *p* = 0.025; from VC to Acb shell: *t* = −2.872, *p* = 0.012; from Acb core to OFC: *t* = 4.066, *p* = 0.001; from Acb shell to OFC: *t* = 3.458, *p* = 0.004) in the right hemisphere. Results of the early-stage rTMS intervention revealed that right nucleus accumbens played the role as a central hub, and VC was a potentially novel rTMS target region during adolescent schizophrenia. Moreover, the EC of right nucleus accumbens shell and orbitofrontal cortex was demonstrated to be a potential biomarker. To our knowledge, this was the first resting-state fMRI study using GCA to assess the deficits of a visual-reward neural pathway and the effectiveness of rTMS treatment in MAM rats. More randomized controlled trials in both animal models and schizophrenia patients are needed to further elucidate the disease characteristics.

## Introduction

Although the mechanisms of schizophrenia remain unclear, altered brain maturational processes can be found in schizophrenia patients, suggesting schizophrenia as a potential neurodevelopmental disease (Armenteros and Davies, 2006; Brent et al., 2013). Adolescence is considered a crucial period of brain development, and schizophrenia diagnosed in this stage is labeled as adolescent-onset schizophrenia (AOS) (Li et al., 2019). An earlier onset age often predicts poorer outcomes (Rotstein et al., 2018); on the other hand, both human and animal models indicate that the symptoms of schizophrenia can be improved in young patients through intervention (Nordentoft et al., 2009; Gomes et al., 2016; Hashimoto, 2019). These models suggest that studying AOS carries a significant clinical value. In order to investigate the mechanisms of schizophrenia and evaluate the effectiveness of early interventions, methylazoxymethanol acetate (MAM)-embryonic day 17 rats, one of the most accepted schizophrenia rat models (Kállai et al., 2017), were chosen to be the AOS rat model.

Resting-state functional magnetic resonance imaging (fMRI) was widely used to investigate the mechanisms of schizophrenia; indicators such as amplitude of low-frequency fluctuation (ALFF) and functional connectivity (FC) were employed to describe neural activities (Zheng et al., 2020). Abnormal ALFF and FC signals between primary sensory cortices (such as visual cortex, VC) and prefrontal cortex (such as orbitofrontal cortex, OFC) were detected in schizophrenia patients as well as in MAM model, thus indicating imbalanced frontal-posterior cerebral activities (Kaneko et al., 2017; Chang et al., 2019; Xia et al., 2019). Moreover, VC was a critical brain region in schizophrenia that could result in various neurodegenerative symptoms like visual hallucinations; deficits of VC-related structural and functional connectivities were found in both schizophrenia patients and first-degree relatives (Maher et al., 2019; Kurtulmus et al., 2020). Considering the neurodevelopmental order ranging from primary cortex to senior cortex, we further speculated that VC was an initial abnormal region during the early stage of schizophrenia, and there might also exist a subcortical region mediating brain activities among primary sensory cortices and prefrontal cortex. It is generally acknowledged that the mechanism of schizophrenia is tightly related to deficit in the reward system (Whitton et al., 2015). The nucleus accumbens (Acb) of striatum plays a central role in reward processing (de Leeuw et al., 2015; McDevitt and Graziane, 2018); moreover, VC and OFC both reveal cortical–striatal connections with striatum, further suggesting that Acb is a central hub connecting VC and OFC (Kuo and Liu, 2019). We assumed that an effective early-stage intervention specifically targeting VC could probably help mitigate the altered network, and the abnormal directional VC–Acb–OFC connection might be a critical biomarker for schizophrenia.

Repetitive transcranial magnetic stimulation (rTMS) is a non-invasive brain stimulation technique used for treating various mental disorders. It has been clinically approved by the U.S. Food and Drug Administration for treatment-resistant depression (George et al., 2013), and one large study also showed rTMS to be effective in schizophrenia patients (Dougall et al., 2015). Moreover, one meta-analysis involving 30 randomized controlled trials (RCT) further suggested that rTMS could relieve the symptoms of schizophrenia (Kennedy et al., 2018). These studies suggested that rTMS has the potential to be an effective intervention. However, the target region of rTMS in schizophrenia was inconsistent between studies (Marzouk et al., 2019). In this study, we took the novel approach of delivering rTMS targeting the VC of MAM rats during adolescence. We predicted significant alteration of the posterior–anterior connection after rTMS treatment.

In order to further clarify the causal connection in the VC–Acb–OFC pathway in MAM rats and to evaluate the effectiveness of rTMS treatment, we used Granger Causality Analysis (GCA) to analyze the effective connectivity (EC) between the regions of interest (ROIs). Granger Causality defined the causality of time series as follows: if preceding time series x could help predict the present time series y better than what preceding time series y could predict about the present time series y itself, then x “Granger caused” y (Granger, 1969; Granger, 1980). GCA has been widely used in resting-state fMRI studies to assess the altered EC of patients within many diseases, such as major depressive disorder (Luo et al., 2020), Parkinson’s disease (Hao et al., 2020), and epilepsy (Jiang et al., 2018).

In summary, the aim of our resting-state fMRI study was to investigate the potential mechanism of schizophrenia and to find an effective early-stage intervention. MAM rats were used to mimic schizophrenia patients. VC was chosen as a novel target region of intervention, and rTMS treatment was delivered during adolescent stage in MAM rats. GCA was applied to demonstrate the causal link between VC, nucleus accumbens core (AcbC), nucleus accumbens shell (AcbSh), and OFC separately in both hemispheres. We aimed (1) to evaluate the abnormal EC from posterior to anterior cerebrum and to find a potential biomarker in schizophrenia and (2) to assess the effectiveness of rTMS intervention on early-stage schizophrenia. We hypothesized significant alteration of EC in the VC–Acb–OFC pathway both in MAM rats and after rTMS treatment.

## Materials and Methods

### Animals

The rats were provided by VITAL RIVER Laboratories, Inc. (Beijing, China). On embryonic day 17, SPF level Sprague–Dawley rats were randomly, blindly divided into MAM groups (MAM-sham group and MAM-rTMS group) and vehicle groups (vehicle-sham group and vehicle-rTMS group). The MAM groups were injected with 22 mg/kg MAM, and the vehicle groups were injected with 0.9% saline solution. Pups were ablactated on postnatal day (PD) 21 and regrouped according to gender; male rats were chosen as the experiment material, with 2–4 per cage under controlled environment (temperature 21 ± 1°C; humidity 30–70%; light/dark cycle 12 h, lights on at 7:00 a.m.) to mimic normal circadian rhythm, with full access to food and water. All experiments strictly followed the guidelines of the Animal Care and Use Committee at the Wuhan Institute of Physics and Mathematics, Chinese Academy of Sciences.

### rTMS Treatment

**FIGURE 1:**
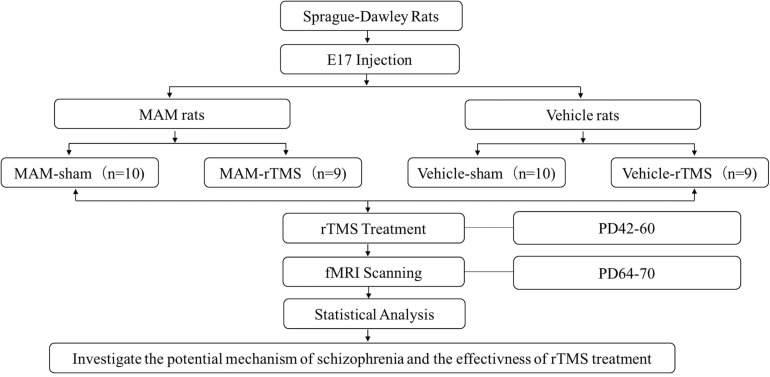
The study design and technical route. E17, embryonic day 17; MAM, methylazoxymethanol acetate; rTMS, repetitive transcranial magnetic stimulation; PD, postnatal day; fMRI, functional magnetic resonance imaging.

The animals in the MAM group (*n* = 19) and Vehicle group (*n* = 19) were randomly divided into the rTMS group and sham group. Hence, the rats were separated into the MAM-rTMS group (*n* = 9), MAM-sham group (*n* = 10), vehicle-rTMS group (*n* = 9), and vehicle-sham group (*n* = 10). For adolescence animals (PD 40–60), stimulation for the rTMS group was carried out through a magnetic stimulator and a ring-shaped animal coil produced by Wuhan Irid Corporation. During the stimulation, the heads of rats were fixed, the coils were tightly adhered to the scalps, and the center of the coils was placed over the anterior interaural line of the skull, targeting VC (see Supplementary Figure 1). In each session, the frequency of the burst train was 10 Hz, with a dosage of 15 trains per day, 60 pulses per train, and 15-s inter-train intervals. Each rat received one session per day, and 2 weeks of treatments were conducted in total. The sham group was placed in the same environment, but the coils were not energized, with only the same frequency of noise stimulations (see Figure 1).

### MRI Acquisition

During adulthood (PD 64–70), a 7.0-T MRI scanner (Bruker Biospin, Ettlingen, German) was used to scan the rat with a rat brain quadrature surface coil for receiving (diameter, 50 mm); the rat was anesthetized with 2% isoflurane. A Rapid Imaging with Refocused Echoes (RARE) sequence was used to position fMRI slices, and an anatomical scan was acquired with the following parameters: repetition time (TR) = 5,000 ms, echo time (TE) = 12 ms, matrix size = 256 × 256, field of view (FOV) = 2.4 × 2.4 cm^2^, slice number = 20, slice thickness = 0.8 mm. An interleaved snapshot echo planar imaging (EPI) was then used with the same localization of anatomical scan to acquire fMRI scan with the following parameters: repetition time (TR) = 1,000 ms, echo time (TE) = 14 ms, matrix size = 64 × 64, field of view (FOV) = 2.4 × 2.4 cm^2^, slice number = 20, slice thickness = 0.8 mm.

### Statistical Analysis

The data were preprocessed using the DPARSF V5.1 Rat module, which is based on SPM8 and the toolbox for Data Processing and Analysis of Brain Imaging (DPABI, rfmri.org/DPABI) (Yan et al., 2016). The anatomical images were first normalized into the standard anatomical rat template (Barrière et al., 2019), and the functional images were then normalized using the parameters generated from the anatomical images. The voxel size of functional images was then processed through the following steps: slice-timing correction, realign, spatial normalization, spatial smooth, removal of the linear trend, 0.01–0.1 Hz band-pass filter, and head-motion correction. After these steps, the preprocessed data were ready for ROI extraction.

The regions of interest (ROIs) were anatomically masked and defined based on the standard anatomical rat template (Barrière et al., 2019). VC, AcbC, AcbSh, and OFC in each hemisphere were separately chosen as the ROIs. In all, eight ROIs were included in our study. The time series of all the voxels in each ROI were extracted and averaged, and the mean value was set as the time series of that ROI.

For each ROI, we calculated the mean and standard deviation values of the time series in four separate groups; then, we made comparisons of the standard deviation values between every two groups to preliminarily confirm the effectiveness of disease and rTMS treatment. We performed two-sample *t*-tests between MAM-sham and vehicle-sham groups, MAM-rTMS and MAM-sham groups, MAM-rTMS and vehicle-rTMS groups, and vehicle-rTMS and vehicle-sham groups. Before each comparison, Levene’s tests were conducted to detect homogeneity of variance. When *p* 0.05, the results were suggested as significant.

Chen’s extended linear autoregressive model was used in our study for GCA (Hamilton et al., 2011). For two ROIs included in our fMRI study, the GCA between two time series x and y could be used to determine the direction among two ROIs (Goebel et al., 2003). For two time series x and y, the auto-regressive models of x and y were defined as below:

$${\text{X}}_{\text{t}}=\sum_{i=1}^{\text{P}}{\text{A}}_{\text{i}}\,{\text{X}}_{\text{t}-i}+{\text{CZ}}_{\text{t}}+\varepsilon_{\text{t}}$$

$${\text{Y}}_{\text{t}}=\sum_{i=1}^{\text{P}}{\text{B}}_{\text{i}}\,{\text{Y}}_{\text{t-i}}+{\text{CZ}}_{\text{t}}+\varepsilon_{\text{t}}$$

The joint-regressive model was defined as below:

$${\text{X}}_{\text{t}}=\sum_{i=1}^{\text{P}}{\text{A}}_{\text{i}}\,{\text{Y}}_{\text{t}-i}+\sum_{i=1}^{\text{P}}{\text{B}}_{\text{i}}\,{\text{X}}_{\text{t}-i}+{\text{CZ}}_{\text{t}}+\mu_{\text{t}}$$

Yt=∑i=1PAXt-i′i+∑i=1PBYt-i′i+CZt′+μt′

Among them, *p* was the regression order, *A_i*and *B_i* were the GC coefficients, and ℰ and μ were the residuals. If A = 0 and ℰ=μ, then x was not the GC of y, and vice versa. Through calculating the GC coefficient between two ROIs, we could judge their GC. Results of right and left hemispheres were calculated separately. The GC of each connectivity was regarded as an independent outcome. We obtained 32 groups of results.

**FIGURE 2:**
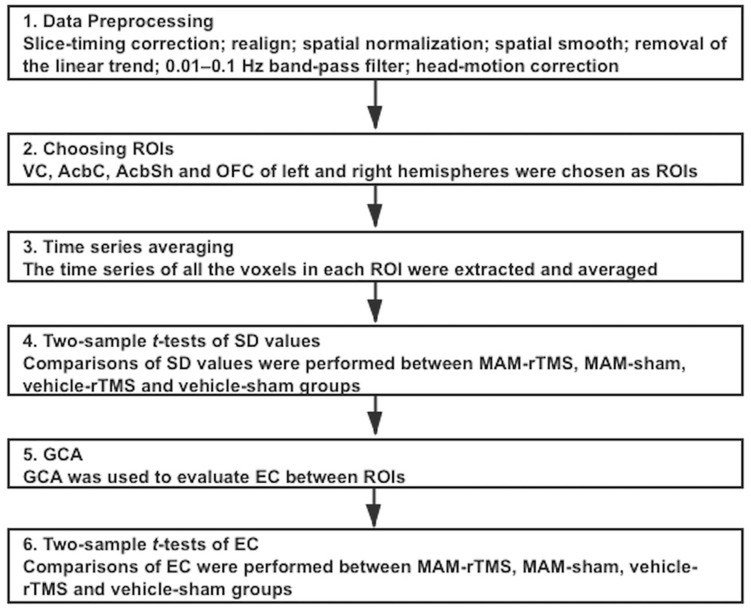
Overview of the procedures of statistical analysis; ROI, regions of interest; VC, Visual Cortex; AcbC, Nucleus Accumbens Core; AcbSh, Nucleus Accumbens Shell; OFC, Orbitofrontal Cortex; SD, standard deviation; MAM, methylazoxymethanol acetate; rTMS, repetitive transcranial magnetic stimulation; GCA, Granger Causality Analysis; EC, Effective Connectivity.

For each connectivity, we made four comparisons to verify our hypotheses. We performed two-sample *t* tests to evaluate the difference in EC between MAM-sham and vehicle-sham groups, MAM-rTMS and MAM-sham groups, MAM-rTMS and vehicle-rTMS groups, and vehicle-rTMS and vehicle-sham groups; comparisons of the left and right hemispheres were separately made. Before each comparison, Levene’s tests were conducted to detect homogeneity of variance. When *p* 0.05, the results were suggested as significant (see Figure 2).

## Results

**FIGURE 3:**
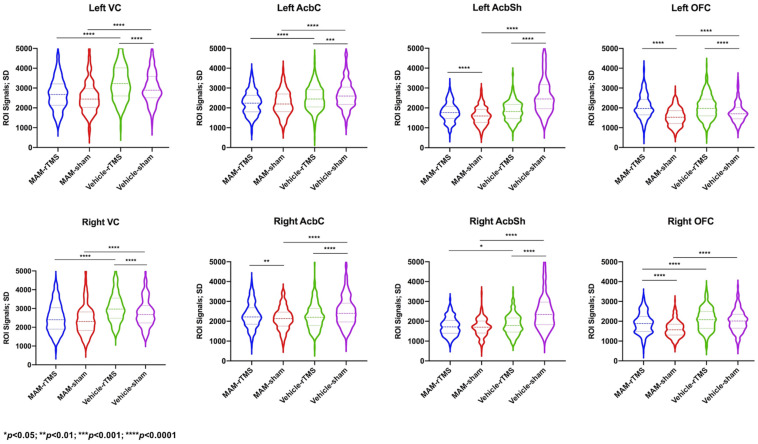
Comparisons of standard deviation values of time series in each region of interest; SD, standard deviation; MAM, methylazoxymethanol acetate; rTMS, repetitive transcranial magnetic stimulation; VC, Visual Cortex; AcbC, Nucleus Accumbens Core; AcbSh, Nucleus Accumbens Shell; OFC, Orbitofrontal Cortex.

**FIGURE 4:**
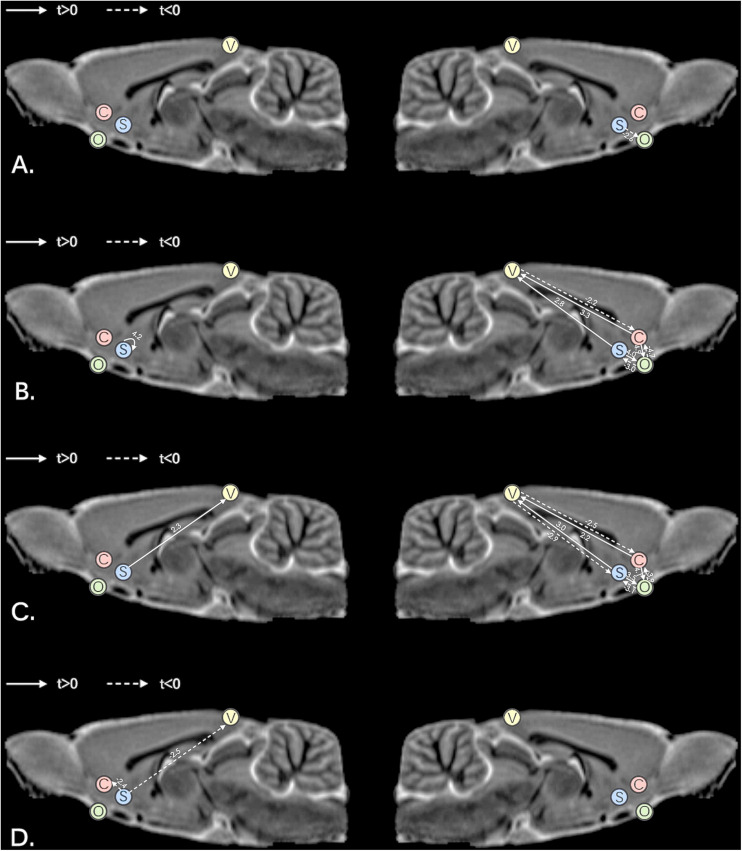
Comparisons of effective connectivity in the left hemisphere and right hemisphere; **(A)** comparisons between MAM-sham and vehicle-sham groups; **(B)** comparisons between MAM-rTMS and MAM-sham groups; **(C)** comparisons between MAM-rTMS and vehicle-rTMS groups; **(D)** comparisons between vehicle-rTMS and vehicle-sham groups; MAM, methylazoxymethanol acetate; rTMS, repetitive transcranial magnetic stimulation; V, Visual Cortex; C, Nucleus Accumbens Core; S, Nucleus Accumbens Shell; O, Orbitofrontal Cortex.

Comparisons between the MAM-sham group, vehicle-sham group, MAM-rTMS group, and vehicle-rTMS group were made. Preliminary comparisons suggested significant differences of time series between both MAM groups and vehicle groups and rTMS groups and sham groups (see Figure 3). Comparisons of EC further confirmed it. The results were shown as below (see Figure 4).

### Comparisons of EC Between MAM-Sham and Vehicle-Sham Groups

Significant differences were found between MAM-sham group and vehicle-sham group. In MAM-sham group, decreased EC from AcbSh to OFC was detected in the right hemisphere (*t* = −2.553, *p* = 0.021), while no significant difference was detected in the left hemisphere (see Table 1 and Figure 4A).

**TABLE 1:** Comparisons of EC between MAM-sham and vehicle-sham groups.

**Hemisphere**	**Source**	**Target**	**Levene’s test *F*(1, 17)**	***P*-value**	**Two-sample *t*-test**	***P-*value**
Left	VC	VC	0.011	0.916	0.805	0.432
		AcbC	0.706	0.412	−0.614	0.547
		AcbSh	0.012	0.915	−0.348	0.732
		OFC	0.207	0.655	−1.198	0.247
	AcbC	VC	0.543	0.471	−0.524	0.607
		AcbC	0.02	0.889	−0.281	0.782
		AcbSh	2.249	0.152	0.621	0.543
		OFC	0.019	0.892	−0.946	0.357
	AcbSh	VC	0.012	0.913	−0.819	0.424
		AcbC	0.678	0.422	−0.666	0.514
		AcbSh	1.413	0.251	−1.938	0.069
		OFC	0.98	0.336	−1.724	0.103
	OFC	VC	0.279	0.604	1.101	0.286
		AcbC	0.244	0.628	−0.587	0.565
		AcbSh	1.246	0.28	−0.14	0.89
		OFC	0.447	0.513	0.24	0.813
Right	VC	VC	1.482	0.24	0.698	0.495
		AcbC	0.297	0.593	−0.845	0.41
		AcbSh	2.432	0.137	0.216	0.832
		OFC	0.712	0.411	−1.286	0.216
	AcbC	VC	0.008	0.93	0.542	0.595
		AcbC	0.168	0.687	0.482	0.636
		AcbSh	2.852	0.109	0.107	0.916
		OFC	0.818	0.378	−1.421	0.173
	AcbSh	VC	2.853	0.109	−0.541	0.595
		AcbC	0.05	0.826	0.474	0.641
		AcbSh	0.093	0.764	−1.076	0.297
		OFC	2.719	0.118	−2.553	0.021*
	OFC	VC	0.382	0.545	0.607	0.552
		AcbC	< 0.001	0.983	0.883	0.389
		AcbSh	2.022	0.173	0.904	0.379
		OFC	2.267	0.151	−0.215	0.833

*p < 0.05.

*EC, Effective Connectivity; MAM, methylazoxymethanol acetate; VC, Visual Cortex; AcbC, Nucleus Accumbens Core; AcbSh, Nucleus Accumbens Shell; OFC, Orbitofrontal Cortex.*

### Comparisons of EC Between MAM-rTMS and MAM-Sham Groups

Compared to the MAM-sham group, the MAM-rTMS group suggested significant differences of EC. In the right hemisphere, post-intervention MAM rats revealed decreased EC from VC to AcbC (*t* = −2.206, *p* = 0.043), from OFC to AcbC (*t* = −4.708, *p* < 0.001), and from OFC to AcbSh (*t* = −3.048, *p* = 0.008). Increased EC was found from AcbC to VC (*t* = 3.339, *p* = 0.004), from AcbC to OFC (*t* = 4.861, *p* < 0.001), from AcbSh to VC (*t* = 2.766, *p* = 0.014), and from AcbSh to OFC (*t* = 4.025, *p* = 0.001). In the left hemisphere, only increased EC from AcbSh to AcbSh (*t* = 4.182, *p* = 0.001) was found after rTMS treatment (see Table 2 and Figure 4B).

**TABLE 2:** Comparisons of EC between MAM-rTMS and MAM-sham groups.

**Hemisphere**	**Source**	**Target**	**Levene’s test *F*(1, 15)**	***P*-value**	**Two-sample *t*-test**	***P*-value**
Left	VC	VC	0.889	0.361	0.682	0.506
		AcbC	7.326	0.016*	−0.39	0.705
		AcbSh	3.376	0.086	0.252	0.805
		OFC	0.005	0.944	1.454	0.167
	AcbC	VC	1.631	0.221	0.851	0.408
		AcbC	1.023	0.328	0.647	0.527
		AcbSh	0.264	0.615	−0.476	0.641
		OFC	0.141	0.713	0.453	0.657
	AcbSh	VC	2.426	0.14	0.166	0.87
		AcbC	0.541	0.473	0.376	0.713
		AcbSh	1.433	0.25	4.182	0.001**
		OFC	1.569	0.23	−0.416	0.684
	OFC	VC	1.598	0.226	−1.627	0.125
		AcbC	1.303	0.271	0.965	0.35
		AcbSh	0.758	0.398	1.544	0.144
		OFC	<0.001	0.999	1.479	0.16
Right	VC	VC	3.214	0.093	0.97	0.347
		AcbC	<0.001	0.992	−2.206	0.043*
		AcbSh	1.6	0.225	−1.887	0.079
		OFC	0.409	0.532	1.179	0.257
	AcbC	VC	0.013	0.912	3.339	0.004**
		AcbC	0.004	0.953	0.151	0.882
		AcbSh	0.086	0.773	−0.3	0.768
		OFC	0.018	0.894	4.861	<0.001***
	AcbSh	VC	0.985	0.337	2.766	0.014*
		AcbC	0.095	0.762	−0.251	0.805
		AcbSh	1.195	0.292	1.726	0.105
		OFC	0.925	0.351	4.025	0.001**
	OFC	VC	3.731	0.073	−1.351	0.197
		AcbC	0.378	0.548	−4.708	<0.001***
		AcbSh	2.186	0.16	−3.048	0.008**
		OFC	2.366	0.145	0.24	0.814

*p < 0.05; **p < 0.01; ***p < 0.001.

*EC, Effective Connectivity; MAM, methylazoxymethanol acetate; rTMS, repetitive transcranial magnetic stimulation; VC, Visual Cortex; AcbC, Nucleus Accumbens Core; AcbSh, Nucleus Accumbens Shell; OFC, Orbitofrontal Cortex.*

### Comparisons of EC Between MAM-rTMS and Vehicle-rTMS Groups

Significant differences were found between the MAM-rTMS group and vehicle-rTMS group. In the right hemisphere, the MAM-rTMS group revealed decreased EC from VC to AcbC (*t* = −2.482, *p* = 0.025), from VC to AcbSh (*t* = −2.872, *p* = 0.012), from OFC to AcbC (*t* = −3.93, *p* = 0.003), and from OFC to AcbSh (*t* = −3.051, *p* = 0.008). Increased EC was found from AcbC to VC (*t* = 2.232, *p* = 0.041), from AcbC to OFC (*t* = 4.066, *p* = 0.001), from AcbSh to VC (*t* = 3.028, *p* = 0.008), and from AcbSh to OFC (*t* = 3.458, *p* = 0.004). In the left hemisphere, only increased EC from AcbSh to VC (*t* = 2.262, *p* = 0.039) was found in the MAM-rTMS group compared to the vehicle-rTMS group (see Table 3 and Figure 4C).

**TABLE 3:** Comparisons of EC between MAM-rTMS and vehicle-rTMS groups.

**Hemisphere**	**Source**	**Target**	**Levene’s test *F*(1, 15)**	***P*-value**	**Two-sample *t*-test**	***P*-value**
Left	VC	VC	0.524	0.48	0.865	0.401
		AcbC	2.255	0.154	−0.867	0.4
		AcbSh	0.092	0.766	−1.711	0.108
		OFC	0.177	0.68	0.5	0.624
	AcbC	VC	0.007	0.933	2.146	0.049
		AcbC	2.138	0.164	0.011	0.991
		AcbSh	0.939	0.348	−1.565	0.139
		OFC	0.053	0.821	0.455	0.655
	AcbSh	VC	0.35	0.563	2.262	0.039*
		AcbC	0.507	0.487	1.972	0.067
		AcbSh	3.851	0.069	0.986	0.34
		OFC	0.127	0.726	−0.291	0.775
	OFC	VC	2.28	0.152	−0.19	0.852
		AcbC	1.129	0.305	−0.037	0.971
		AcbSh	1.65	0.219	−0.557	0.586
		OFC	0.732	0.406	0.52	0.611
Right	VC	VC	0.468	0.504	0.747	0.467
		AcbC	0.013	0.911	−2.482	0.025*
		AcbSh	0.894	0.359	−2.872	0.012*
		OFC	0.017	0.899	−0.541	0.596
	AcbC	VC	1.9	0.188	2.232	0.041*
		AcbC	0.56	0.466	0.82	0.425
		AcbSh	0.003	0.958	−1.327	0.204
		OFC	0.01	0.92	4.066	0.001**
	AcbSh	VC	0.539	0.474	3.028	0.008**
		AcbC	0.073	0.791	0.807	0.432
		AcbSh	0.042	0.841	0.921	0.372
		OFC	0.764	0.396	3.458	0.004**
	OFC	VC	3.371	0.086	1.246	0.232
		AcbC	4.577	0.049*	−3.93	0.003**
		AcbSh	1.022	0.328	−3.051	0.008**
		OFC	0.384	0.545	−0.254	0.803

*p < 0.05; **p < 0.01.

*EC, Effective Connectivity; MAM, methylazoxymethanol acetate; rTMS, repetitive transcranial magnetic stimulation; VC, Visual Cortex; AcbC, Nucleus Accumbens Core; AcbSh, Nucleus Accumbens Shell; OFC, Orbitofrontal Cortex.*

### Comparisons of EC Between Vehicle-rTMS and Vehicle-Sham Groups

Compared to the vehicle-sham group, the vehicle-rTMS group suggested significant differences of EC. In the vehicle-rTMS group, decreased EC from AcbSh to VC (*t* = −2.489, *p* = 0.023) as well as AcbC (*t* = −2.418, *p* = 0.027) was detected in the left hemisphere, while no significant difference was detected in the right hemisphere (see Table 4 and Figure 4D).

**TABLE 4:** Comparisons of EC between vehicle-rTMS and vehicle-sham groups.

**Hemisphere**	**Source**	**Target**	**Levene’s test *F*(1, 17)**	***P*-value**	**Two-sample *t*-test**	***P*-value**
Left	VC	VC	0.003	0.955	0.513	0.615
		AcbC	0.478	0.498	−0.529	0.603
		AcbSh	1.363	0.259	0.713	0.485
		OFC	0.014	0.907	−0.029	0.977
	AcbC	VC	2.684	0.12	−1.24	0.232
		AcbC	0.565	0.463	0.24	0.813
		AcbSh	0.03	0.864	1.578	0.133
		OFC	< 0.001	0.983	−0.926	0.367
	AcbSh	VC	2.842	0.11	−2.489	0.023*
		AcbC	0.607	0.447	−2.418	0.027*
		AcbSh	4.497	0.049*	0.562	0.586
		OFC	0.071	0.793	−1.541	0.142
	OFC	VC	0.042	0.841	−0.037	0.971
		AcbC	0.542	0.472	0.22	0.829
		AcbSh	0.598	0.45	1.493	0.154
		OFC	0.125	0.728	1.698	0.108
Right	VC	VC	0.049	0.827	0.722	0.48
		AcbC	0.406	0.533	−0.641	0.53
		AcbSh	4.109	0.059	0.453	0.656
		OFC	1.824	0.195	0.05	0.96
	AcbC	VC	1.997	0.176	0.237	0.815
		AcbC	0.235	0.634	−0.303	0.766
		AcbSh	4.275	0.054	0.922	0.369
		OFC	0.877	0.362	−0.892	0.385
	AcbSh	VC	0.008	0.93	−1.283	0.217
		AcbC	0.026	0.875	−0.736	0.472
		AcbSh	1.856	0.191	−0.232	0.819
		OFC	2.585	0.126	−1.987	0.063
	OFC	VC	0.597	0.45	−1.337	0.199
		AcbC	1.845	0.192	−0.469	0.645
		AcbSh	4.139	0.058	0.45	0.658
		OFC	0.404	0.533	0.286	0.778

*p < 0.05.

*EC, Effective Connectivity; rTMS, repetitive transcranial magnetic stimulation; VC, Visual Cortex; AcbC, Nucleus Accumbens Core; AcbSh, Nucleus Accumbens Shell; OFC, Orbitofrontal Cortex.*

## Discussion

In the current study, we found an alteration of EC in the right VC–Acb–OFC pathway, which characterized both the potential mechanism of schizophrenia and the effectiveness of a novel rTMS treatment through the MAM rat model; moreover, right AcbSh-OFC EC was suggested as a potential biomarker for both diagnosis and early-stage intervention of schizophrenia. Results revealed significant lateralization in the right hemisphere.

### VC Was Indicated as a Potential rTMS Target Region During Early Intervention

To our knowledge, few studies have characterized VC as an rTMS target region (Thut et al., 2003; Lang et al., 2007; Rafique and Steeves, 2020) and none of them were schizophrenia-related; however, one study suggested that accelerated rTMS significantly changed glutamate and γ-aminobutyric acid (GABA)+ concentration and had great capability for treating visual disorders (Rafique and Steeves, 2020). This implies that rTMS treatment on VC substantially improved schizophrenia symptoms. We predicted that dysfunction of VC might happen in early stages of schizophrenia, resulting in primary cortex deficits; then, it influenced senior cortex, such as OFC, indirectly through Acb. After delivering rTMS treatment on VC during adolescence, the EC from VC to Acb decreased, and in compensation lead to increased EC from Acb to OFC. Likewise, Kaneko et al. found altered FC between VC and striatum, and OFC in adolescent MAM rats (Kaneko et al., 2017), further providing a strong basis for our hypothesis. Our results indicate that VC has the potential of becoming an rTMS target region during early-stage schizophrenia, indicating that more studies on both human and animal models are needed.

### Right Acb Played the Role of a Central Hub Among Posterior–Anterior EC in rTMS Treatment

After rTMS treatment, significant alteration was found in the right hemisphere; AcbC and AcbSh together revealed significant alteration of EC with VC as well as OFC, thus indicating right Acb as an important linkage between cortices during intervention. It has been clinically approved for deep brain stimulation (DBS) targeting Acb to treat obsessive–compulsive disorder (OCD), and one resting-state fMRI study suggested that it could normalize Acb-related connectivity in reward circuits (Figee et al., 2013; Park et al., 2019). We postulate that similar effects could exist in our study. The cascade between VC and Acb helps amplify the intervention effect, coupling the transmission from Acb to OFC; meanwhile, compensatory effects inversely affected the whole neural circuit, resulting in complicated alteration of EC between VC, Acb, and OFC. Consistent with our theory, the anatomical projection from cortices to striatum in the visual corticostrial loop (Kuo and Liu, 2019) demonstrated the existence of the visual reward-related neural network; meanwhile, altered FC was found in both VC-Acb connectivity and Acb-OFC connectivity in schizophrenia patients (Fischer et al., 2014). Our results further clarified the causal link within the connectivities. In all, right Acb played the role of a central hub among the EC between posterior and anterior cerebrum during the intervention, and our study demonstrated for the first time the casual relationship between Acb and VC in MAM rats.

### Right AcbSh-OFC EC Was Demonstrated as a Substantial Biomarker in Schizophrenia

The right AcbSh-OFC EC showed significant difference between both MAM-sham and vehicle-sham groups and MAM-rTMS and MAM-sham groups; according to our results, we suggest that schizophrenia decreased the causal link from AcbSh to OFC, and early-stage rTMS treatment increased it compensately. As a subregion of the Acb, AcbSh is mostly made up of GABAergic medium spiny neurons (MSNs) (McDevitt and Graziane, 2018; Castro and Bruchas, 2019) and participates in motivation as well as emotional processing (Park et al., 2019). Both AcbSh and OFC were core regions of the mesocorticolimbic dopamine system, and previous studies revealed their synchronized activation during fMRI acquisition in reward processing (Carlson et al., 2011). Contrary to the generally accepted theory that impaired top-down control of frontal-striatum connectivity leads to deficits in schizophrenia (Richter et al., 2015), our study indicates that a reverse bottom-up dysfunction from AcbSh to OFC resulted in the unregulated reward system of MAM rats. Moreover, since the mechanism of rTMS was dopaminergic-involved, and TMS could induce elevation of extracellular dopamine and glutamate in Acb (Zangen and Hyodo, 2002; Aleman, 2013), we suggest that the decreased EC was normalized after early-stage rTMS treatment. A previous diffusion tensor imaging (DTI) study in schizophrenia reported decreased connectivity between AcbC and OFC, while no significant change in AcbSh was found (Amodio et al., 2018). We creatively observed the altered AcbSh-OFC EC in MAM rats and successfully normalized it after early-stage rTMS treatment. Altogether, the right AcbSh-OFC EC was demonstrated as a substantial biomarker in schizophrenia.

There were several limitations in our resting-state fMRI study. Firstly, no significant difference of EC from VC to Acb was detected between the MAM-sham group and the vehicle-sham group. We hypothesized the reason for this was that in contrast to senior cortex OFC, VC was a primary sensory cortex and Acb was a subcortical region, both of which undertook fundamental functions in brain. During neurodevelopment, deficits from VC to Acb occurred in the early stage of schizophrenia, while the Acb-OFC EC progressed in adulthood. As a consequence, we failed to detect significantly different VC-Acb EC in our results. Future studies targeting early diagnosis could take our hypothesis into consideration and explore the potential alteration of this connectivity. Secondly, GCA was used to reveal Granger causal links in the pathway, and the results suggested synchronization of time series between ROIs; however, the precise mechanism remains unclear. More related studies are needed in the future to verify our results. Finally, significant lateralization was found after rTMS treatment. Although our intervention chose the whole VC as the target region, rTMS treatment mainly came into effect in the right hemisphere. This might be attributed to the inhibitory effect of rTMS in the right side, and partly explained why decreased VC-Acb EC was found after intervention (Lefaucheur et al., 2014). Future studies exploring the mechanism of rTMS treatment might help clarify our postulates.

## Conclusion

We found a critical right VC–Acb–OFC pathway in the MAM rat model using GCA, indicating that abnormal posterior–anterior EC might be the substantial mechanism of schizophrenia; rTMS treatment was suggested as an effective early-stage intervention, among which right Acb played the role of a central hub, and VC showed its potential of becoming a novel rTMS target region during adolescent schizophrenia. Moreover, right AcbSh-OFC EC was shown to be a substantial biomarker. To our knowledge, this was the first resting-state fMRI study using GCA to assess the deficits of a visual-reward neural pathway and the effectiveness of rTMS treatment in MAM rats. More high-quality, multiple-center randomized controlled trials in both animal models and schizophrenia patients are needed.

## Data Availability Statement

The raw data supporting the conclusions of this article will be made available by the authors, without undue reservation.

## Ethics Statement

The animal study was reviewed and approved by the Animal Care and Use Committee at the Wuhan Institute of Physics and Mathematics, Chinese Academy of Sciences.

## Author Contributions

FW: conceptualization. HG, DS, JY, HW, and YZ: methodology. CP: software. HG, DS, and JY: validation. YX, CP, CL, and PZ: formal analysis. HG, DS, and JY: investigation. JW: resources. CL: data curation. YX: writing—original draft preparation. YX, YZ, JFW, and FW: writing—review and editing. YX and JFW: visualization. JW, XZ, and FW: supervision and project administration. DS and FW: funding acquisition. All authors contributed to the article and approved the submitted version.

## Conflict of Interest

The authors declare that the research was conducted in the absence of any commercial or financial relationships that could be construed as a potential conflict of interest.

## Abbreviations

Acb: nucleus accumbens
AcbC: nucleus accumbens core
AcbSh: nucleus accumbens shell
ALFF: amplitude of low-frequency fluctuation
AOS: adolescent-onset schizophrenia
EC: effective connectivity
FC: functional connectivity
GCA: Granger Causality Analysis
MAM: methylazoxymethanol acetate
VC: visual cortex
OFC: orbitofrontal cortex
PD: postnatal day
ROI: regions of interest.
