# Supplementary figures and images for: Early-Stage Repetitive Transcranial Magnetic Stimulation Altered Posterior–Anterior Cerebrum Effective Connectivity in Methylazoxymethanol Acetate Rats
Source: Front Neurosci. 2021 May 21;15:652715. doi: 10.3389/fnins.2021.652715 (PMC8176023; doi:10.3389/fnins.2021.652715)

Supplementary Material


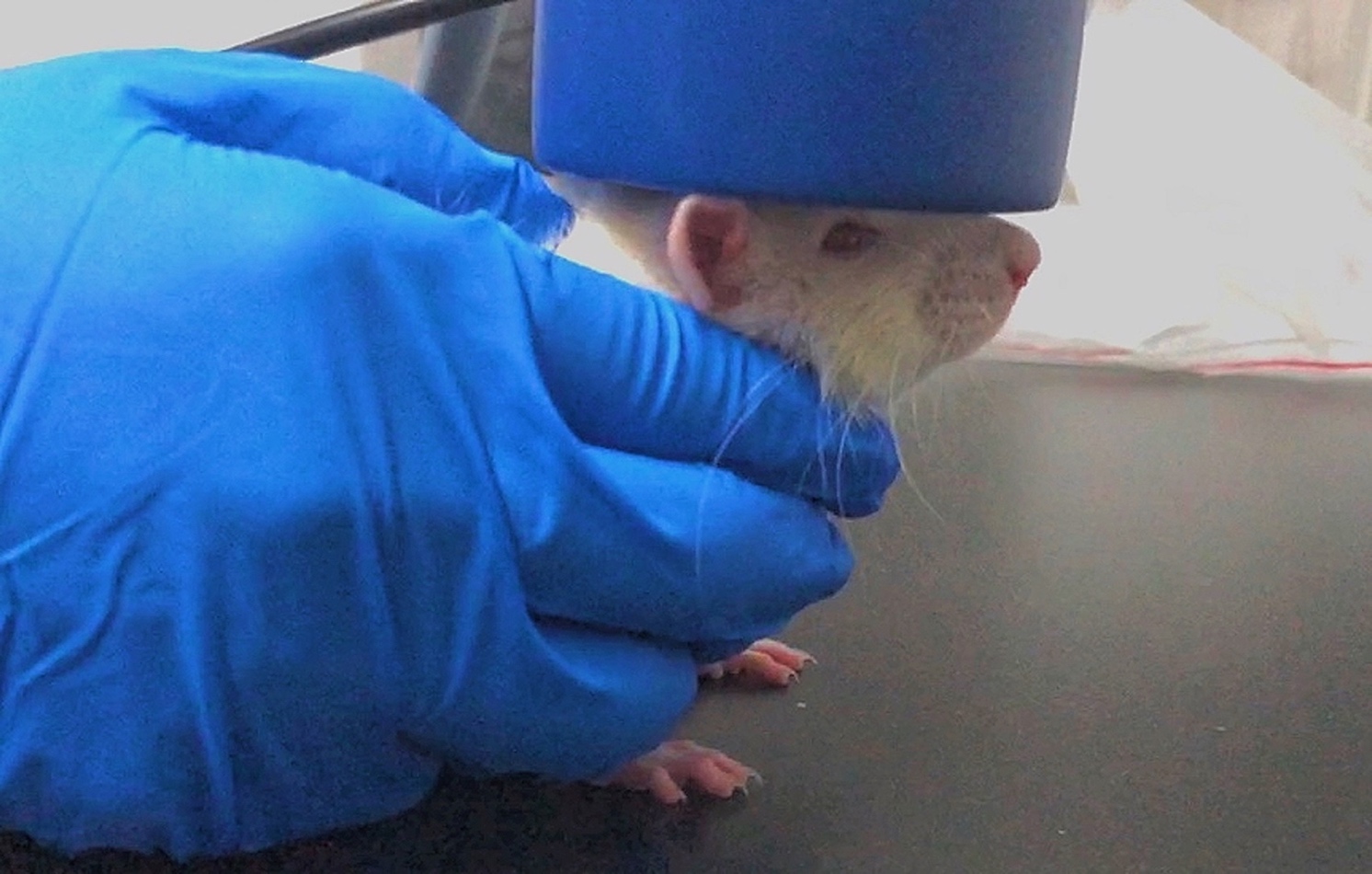


**Supplementary Figure 1.** One rat under rTMS treatment

Supplement: Supplementary file 1 [file Table_1.DOCX]
